# Adherence Definitions, Measurement Modalities, and Psychometric Properties in HIV, Diabetes, and Nutritional Supplementation Studies: A Scoping Review

**DOI:** 10.2147/PPA.S498537

**Published:** 2025-02-11

**Authors:** Julia Burleson, Daryl E Stephens, Rajiv N Rimal

**Affiliations:** 1 Department of Health, Behavior and Society, Bloomberg School of Public Health, Johns Hopkins University, Baltimore, MD, USA

**Keywords:** validity, reliability, medication adherence

## Abstract

Measuring adherence has been a priority for researchers to help inform effective care for patients regularly consuming medications for chronic conditions. As a widely accepted “gold standard” adherence measure or operational definition does not exist, studies measure adherence using different modalities, which may lead to different conclusions about adherence patterns. The purpose of the scoping review was to identify modalities used to measure adherence to HIV medication, diabetes medication, and nutritional supplementation and explore the variation in adherence definitions, measurement modalities, and psychometric properties being reported across studies. Comprehensive searches were performed in PubMed, Scopus, and PsycINFO from January 2012 to January 2022. We included studies reporting psychometric properties of adherence/compliance to HIV medication, diabetes medication, or nutritional supplements. In total, we included 88 studies in the review. The 8-item Morisky Medication Adherence Scale (MMAS-8) was the most frequently used self-reported measure. We found almost no relationship between country income level and triangulation levels. The operational definition of adherence fell into four categories: numerical, dichotomous, ranked ordinal, and undefined. The amount of variation in an adherence definition category within a modality depended on whether the measures within the modality could be assessed numerically and whether widely accepted cutoffs existed for the measure. Across studies, 46 (52%) reported both validity and reliability, 28 (31%) reported validity only, and 14 (16%) reported reliability only. Fourteen types of validity and six types of reliability were identified across the studies. Measuring adherence accurately and reliably continues to be a challenge for research in HIV, diabetes, and nutritional supplementations. When reporting adherence measurements, we suggest including adherence results from multiple measures and modalities, presenting adherence results numerically, and reporting multiple types of validity and reliability.

## Background

Suboptimal adherence to medications often hinders effective care for patients who regularly consume medications over prolonged periods.^1^ High levels of suboptimal adherence and nonadherence can lead to increased morbidity and mortality across illnesses.^2^ Diabetes, HIV, and micronutrient deficiencies are three chronic public health conditions that can be managed or ameliorated by adhering to medication treatments. We explored HIV and diabetes due to the large body of literature on adherence within the health topics. We included nutrition because this paper is part of a larger project on adherence to nutritional supplements.

Current estimates indicate that adherence to treatment remains low for all three conditions. While 38.4 million people worldwide lived with HIV in 2021, on average, only about 60% adhered to antiretroviral therapy guidelines.^3–7^ Adherence can prevent viral drug resistance, slow HIV progression, and reduce the risk of HIV transmission.^8^ Worldwide, 537 million adults lived with diabetes in 2021, and treatment adherence ranged from 38.5 to 93.1%.^9^,^10^ Adherence to diabetes treatments such as insulin or oral hypoglycemic medication can help control hyperglycemia and prevent vision loss, limb amputations, and myocardial infarction.^11^ Finally, one in three people around the world is estimated to have a micronutrient deficiency.^12^ Data on adherence to micronutrient supplements largely focuses on adherence to iron supplements in women of reproductive age, especially pregnant women. A study exploring iron folic acid adherence for pregnant women in 22 countries with high burdens of undernutrition found that only 8% of pregnant women adhered to the ideal iron folic acid supplementation schedule.^13^ While the effects of micronutrient deficiencies depend on the micronutrient, they can cause weakness, brain damage, and increase the risk of severe infections.^14–17^

The World Health Organization defines adherence as “the extent to which a person’s behavior (including medication-taking) corresponds with agreed recommendations from a health care provider”.^2^ Most researchers agree on the conceptual definition of adherence, but there is no consensus on its operational definition, mostly because a widely accepted “gold standard” adherence measure or operational definition does not exist. Studies measure adherence using different modalities (including self-reports and blood samples), which may lead to different conclusions about adherence patterns.

We define *modalities* of adherence measurement as channels used to assess adherence or the way that adherence information is collected. We define *measures* as the means of data collection that can fall within a modality. For example, questionnaires, pill counts (self-reported), in-person interviews, and telephone interviews are measures under the self-report modality. Adherence measures also fall under two categories: direct and indirect measures. Direct measures assess the concentration of a medicine in the body. Indirect measures do not assess the amount of the medication in the body but measure something approximating the amount of the medication ingested. For example, self-reported questionnaires may ask about medication-taking habits to approximate the concentration of medications in participants.

Multiple operational definitions and measurement modalities of adherence can pose research challenges such as inconsistent results and conclusions. When different adherence measurement methods are used, comparing results across studies and identifying erroneous results can be more difficult. However, because there is not a commonly agreed upon gold standard to assess adherence, triangulation may be the most viable strategy for assessing and reporting adherence. Triangulation (ie, relying on multiple measures or modalities) can increase the rigor of research findings by limiting the impact of bias or error associated with any one method and demonstrating similar findings across different adherence measurement methods.^18^

The purpose of this scoping review is to identify methods and modalities used to measure adherence. We focus specifically on HIV medication, diabetes medication, and nutritional supplementation and explore the variation in adherence definitions, measurement modalities, and psychometric property reporting across studies.

## Methods

We conducted this scoping review largely following the methodology published by Peters et al.^19^ The Preferred Reporting Items for Systematic Reviews and Meta-Analyses extension for scoping review (PRISMA-ScR) reporting guideline was used (Supplementary Material 1 PRISMA-ScR Checklist).^20^

We searched three databases for studies on psychometric properties of adherence measurements: PubMed, PsycINFO, and Scopus. The search strategy included three sections: treatment adherence/compliance terms, study area terms (ie, HIV, diabetes, or nutritional supplementation terms), and psychometric terms (Supplementary Material 2 Search Strategy). The PubMed search included both MeSH and text word search field tabs. The PsycINFO and Scopus searches included title, abstract, and keyword search field tabs. We included both “adherence” and “compliance” as search terms in our strategy because the terms are often used interchangeably.^21–23^ The search ran in May 2022, and we uploaded all papers resulting from our search strategy onto Covidence.

### Inclusion and Exclusion Criteria

We included only peer-reviewed, primary research studies written in English. We limited our review to studies published between January 1, 2012, to January 1, 2022, those that reported psychometric properties of adherence/compliance (ie, validity, reliability) and had adherence or compliance to HIV or diabetes medications or nutritional supplements as a behavioral outcome. However, we included glucose monitor articles if they met all other inclusion criteria except having an adherence/compliance behavioral outcome. Many anti-diabetic drugs influence glucose levels, so we included glucose monitors as they have been used as proxies for adherence.

We excluded studies that explored adherence or compliance but did not present data on the psychometric properties of adherence or compliance measures. For example, some studies reported the level of nutritional supplementation adherence in a community and identified individual- and community-level factors that were associated with nutritional supplementation adherence such as education or income. However, we excluded these studies because they did not report any psychometric data on their adherence data.

### Selection Process

All studies from the database searches were uploaded into Covidence, which automatically deleted duplicate studies. One reviewer then manually screened the studies in Covidence for duplicates. Two reviewers then performed initial screenings on ten articles based on an article’s title, abstract, and keywords. The reviewers had 90% agreement. After resolving the disagreement through consensus, one reviewer completed the title, abstract, keyword, and full-text screening for the rest of the articles. Occasionally, the second reviewer assisted in article screening when the first reviewer was unsure if the article should be included.

### Data Charting Process

We developed an extraction form in Covidence to facilitate the extraction process. Two members of the research team piloted the form on five articles to ensure information was captured consistently and completely. We did not revise the extraction table. The rest of the data were single-extracted, and two research team members reviewed the completed extraction table.

### Data Items

The extracted variables included: general article information (title, authors, year of publication, funding, possible conflicts of interest), study characteristics (aims, study topic area, eligibility criteria, country, country income level), intervention characteristics (adherence measure type, adherence measure name, adherence definitions, validity calculations, reliability calculations), population characteristics (sample size, age, sex), study findings and conclusions.

### Synthesis of Results

We explored the frequency and type of modality and measure used by health topic (ie, HIV, diabetes, and nutrition). In each study, the researchers only looked at the psychometric properties of adherence for one health topic. We examined differences in adherence definition categories by measures and modalities. We analyzed triangulation of adherence modalities and measures by country income group. Finally, we looked at the distribution of validity and reliability data by health topic.

## Results

**Figure 1:**
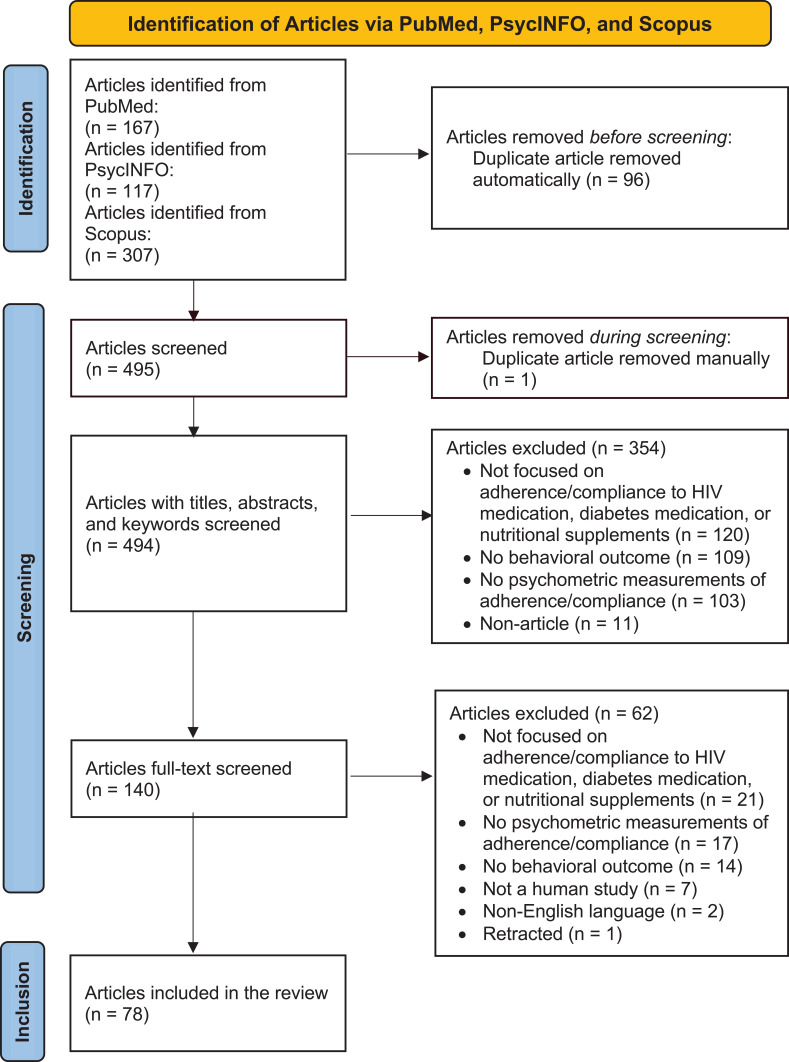
PRISMA ScR Flowchart..

**Notes:** PRISMA figure adapted from Liberati A, Altman D, Tetzlaff J, et al. The PRISMA statement for reporting systematic reviews and meta-analyses of studies that evaluate health care interventions: explanation and elaboration. Journal of clinical epidemiology. 2009;62(10). Creative Commons.^100^

The review identified 591 articles across PubMed, PsycINFO, and Scopus that met the inclusion criteria. After removing duplicates, we screened titles, abstracts, and keywords for 494 articles and excluded 354 articles, mainly because they did not focus on adherence or compliance to HIV or diabetes medications or nutritional supplements. After a full-text screening of 140 articles, we excluded 62 more articles. In total, we included 78 articles in the review, which corresponded to 88 studies as some articles contained multiple studies (Table 1). The PRISMA-ScR flowchart (Figure 1) shows the screening process.

**Table 1:** Overview of Study Characteristics

Study	Health Topic	Country	Modalities	Measures	Adherence Definition Category	Validity/Reliability Types
Teshome et al 2018^24^	Nutrition	Kenya	(1) Blood sample (2) Electronic record (3) Pill count (non-self-reported) (4) Self-reports	(1) Hemoglobin concentration (2) Medication event monitoring system (3) Sachet count (4) Self-reporting sheet	(1) N/A (2) Dichotomous (3) Dichotomous (4) Dichotomous	Uncategorized Validity, Uncategorized Reliability
Agala et al 2020^25^	HIV	Ethiopia	(1) Self-reports	(1) Simplified Medication Adherence Questionnaire	(1) Dichotomous	Internal Consistency Reliability, Concurrent Validity
Agot et al 2015^26^	HIV	Kenya, South Africa, and Tanzania	(1) Blood sample (2) Self-reports (3) Pill count (non-self-reported)	(1) Plasma and intracellular drug concentration (2) In-person interview (3) Pill count	(1) Ranked ordinal (2) Ranked ordinal (3) Dichotomous	Positive Predictive Value
Amico et al 2014^27^	HIV	Peru, Brazil, Ecuador, the United States, Thailand, and South Africa	(1) Blood sample (2) Pill count (non-self-reported) (3) Self-reports (4) Pharmacy record (5) Self-report	(1) TFV-DP concentration (2) In-clinic pill count (3) In-person interview (4) Medication possession ratio (5) Computer-assisted self-interview	(1) Dichotomous (2) Ranked ordinal (3) Ranked ordinal (4) Ranked ordinal (5) Ranked ordinal	Criterion Validity
Berg et al 2012^28^	HIV	United States	(1) Blood sample (2) Self-reports (3) Self-reports (4) Self-reports (5) Self-reports (6) Self-reports	(1) Viral load (2) Rating questionnaire (3) Frequency questionnaire (4) Percent questionnaire (5) Visual analog scale (6) Community Programs for Clinical Research on AIDS	(1) N/A (2) Dichotomous (3) Dichotomous (4) Dichotomous (5) Dichotomous (6) Dichotomous	Construct Validity, Inter-Instrument Reliability
Bucek et al 2020^29^	HIV	United States	(1) Blood sample (2) Self-reports	(1) Viral load (2) Pill count	(1) Dichotomous (2) Numerical	Uncategorized Reliability
Bulgiba et al 2013^30^	HIV	Malaysia	(1) Blood sample (2) Self-reports	(1) Therapeutic drug monitoring (2) Adult AIDS Clinical Trials Group Adherence questionnaire	(1) Numerical (2) Numerical	Sensitivity, Specificity, Negative Predictive Value, Positive Predictive Value
Castillo-Mancilla et al 2015^31^	HIV	United States	(1) Pharmacy record (2) Blood sample (3) Blood sample	(1) Average days between pharmacy refills (2) Dried blood spots (3) Peripheral blood mononuclear cells	(1) Numerical (2) Dichotomous (3) Dichotomous	Uncategorized Reliability
Chai et al 2022^32^	HIV	United States	(1) Pill count (non-self-reported) (2) Blood sample (3) Electronic record	(1) Pill count (2) Dried blood spot (3) Digital pill system	(1) Numerical (2) Dichotomous (3) Numerical	Uncategorized Reliability
Da et al 2018^33^	HIV	China	(1) Blood sample (2) Self-reports (3) Self-reports (4) Self-reports (5) Self-reports	(1) Viral load (2) One-month days missed adherence questionnaire (3) One-month days taken adherence questionnaire (4) 3-day adherence questionnaire (5) Weekend adherence questionnaire	(1) Dichotomous (2) Dichotomous (3) Dichotomous (4) Dichotomous (5) Dichotomous	Sensitivity, Specificity, Inter-Instrument Reliability, Criterion Validity
Desmond et al 2015^34^	HIV	South Africa	(1) Blood sample (2) Self-reports (3) Pharmacy record	(1) Plasma concentration (2) Maternal verbal reports (3) Pharmacy returns	(1) Dichotomous (2) Dichotomous (3) Numerical	Specificity, Sensitivity, Negative Predictive Value, Positive Predictive Value
Dima et al 2013^35^	HIV	Romania	(1) Pill count (non-self-report) (2) Blood sample (3) Medical record (4) Self-reports	(1) Unannounced pill counts (2) Viral load (3) Doctor’s assessment (4) Cuestionario para la Evaluacion de la Adhesion al Tratamiento Antirretroviral en Personas con Infeccion por VIH y Sida	(1) Ranked ordinal (2) Dichotomous (3) Ranked ordinal (4) Dichotomous	External Criterion Validity, Internal Consistency Reliability
Dowshen et al 2013^36^	HIV	United States	(1) Self-reports (2) Self-report	(1) Visual analog scale (2) Interactive text message response	(1) Numerical (2) Numerical	Uncategorized Validity
Fredericksen et al 2014^37^	HIV	United States	(1) Pill count (non-self-reported) (2) Self-reports	(1) Unannounced home-based pill counts (2) Unannounced phone-based pill counts	(1) Numerical (2) Numerical	Inter-Instrument Reliability
Haberer et al 2012^38^	HIV	United States	(1) Blood sample (2) Pill count (non-self-reported) (3) Electronic record	(1) CD4 count (2) Unannounced pill count (3) Med-eMonitor	(1) Dichotomous (2) Numerical (3) Numerical	Uncategorized Validity
Hettema, Hosseinbor, and Ingersoll 2012^39^	HIV	United States	(1) Electronic record (2) Self-reports	(1) Interactive voice response system (2) Timeline follow back	(1) Dichotomous (2) Dichotomous	Inter-Instrument Reliability
Holstad et al 2019^40^	HIV	United States	(1) Self-reports (2) Electronic record	(1) Pill count participant survey (2) Picture Pill Count Scoring Instrument	(1) N/A (2) N/A	Uncategorized Validity, Internal Consistency Reliability, test-retest reliability
Johnston et al 2019^41^	HIV	South Africa	(1) Electronic record (2) Hair sample (3) Blood sample	(1) Electronic adherence monitoring device (2) Hair efavirenz concentrations (3) Blood efavirenz concentrations	(1) N/A (2) N/A (3) N/A	Uncategorized Reliability
Kagee and Nel 2012^42^	HIV	South Africa	(1) Blood sample (2) Self-reports	(1) Viral load (2) Study questionnaire	(1) Dichotomous (2) Numerical	Internal Consistency Reliability, Uncategorized Reliability
Kelly et al 2013^43^	HIV	Sierra Leone	(1) Pill count (non-self-report) (2) Self-reports (3) Self-reports (4) Self-reports	(1) Unannounced pill counts (2) Visual analog scale (3) Adult AIDS Trial Group questionnaire (4) 7-day adherence measure questionnaire	(1) Numerical (2) Numerical (3) Numerical (4) Numerical	Criterion Validity, Uncategorized Reliability
Kerr et al 2012^44^	HIV	Thailand	(1) Blood sample (2) Self-reports (3) Self-reports	(1) Viral load (2) Visual analog scale (3) Center for Adherence Support Evaluation adherence index	(1) Dichotomous (2) Dichotomous (3) Dichotomous	Specificity, Sensitivity
Mariani et al 2020^45^	HIV	Brazil	(1) Blood sample (2) Blood sample	(1) Abbott RealTime HIV-1 viral load assay (2) The mPIMA HIV-1/2 viral load plasma test	(1) N/A (2) N/A	Sensitivity, Specificity, Uncategorized Reliability
Mugisha 2012^46^	HIV	Brazil, UK, Ireland, Spain, Italy, Australia, Uganda, South Africa	(1) Self-reports (2) Blood sample (3) Blood sample	(1) Study-specific questionnaire (2) Viral load (3) Mean corpuscular volume	(1) N/A (2) Dichotomous (3) N/A	Negative Predictive Value, Positive Predictive Value
Pellowski, Kalichman, and Finitsis 2015^47^	HIV	United States	(1) Pill count (non-self-report) (2) Blood sample (3) Self-reports (4) Self-reports	(1) Unannounced pill counts (2) Viral load (3) Computerized single item rating scale (4) Single item rating scale telephone interview	(1) Numerical (2) Dichotomous (3) Numerical (4) Numerical	Concurrent Validity, Criterion Validity, Predictive Validity, Test-Retest Reliability
Rekić et al 2013^48^	HIV	Italy, Norway, France	(1) Other	(1) Bilirubin–atazanavir nomogram	(1) Dichotomous	Negative Predictive Value, Positive Predictive Value, Sensitivity, Specificity
Simoni et al 2014^49^	HIV	United States	(1) Electronic record (2) Blood sample (3) Self-reports	(1) Electronic drug monitor (2) Viral load (3) Self-reported 3-day antiretroviral therapy adherence questionnaire	(1) Numerical (2) Dichotomous (3) Numerical	Uncategorized Reliability
Smith et al 2016^50^	HIV	South Africa	(1) Blood sample (2) Pill count (non-self-reported)	(1) Viral load (2) In-clinic pill count	(1) Dichotomous (2) Dichotomous	Sensitivity, Specificity
Stalter et al 2021^51^	HIV	Uganda, Kenya	(1) Blood sample (2) Urine sample	(1) Liquid chromatography-mass spectrometry assay (2) Enzyme-linked immunosorbent assay	(1) Dichotomous (2) Dichotomous	Sensitivity, Specificity, Uncategorized Reliability
Sun et al 2017^52^	HIV	China	(1) Self-reports (2) Self-reports (3) Self-reports (4) Self-reports (5) Self-reports	(1) Community Programs for Clinical Research on AIDS Antiretroviral Medication Self-Report (2) Social Support Raring Scale (3) 4-item Morisky Scale (4) Chinese HIV Treatment Adherence Self-Efficacy Scale (5) Visual analog scale	(1) Numerical (2) N/A (3) N/A (4) Numerical (5) Numerical	Content Validity, Convergent Validity, Internal Consistency Reliability, Test-Retest Reliability
Tolley et al 2018^53^	HIV	South Africa	(1) Self-reports	(1) Development of Measures of Adherence	(1) N/A	Content Validity, Internal Consistency Reliability
Usitalo et al 2014^54^	HIV	United States	(1) Blood sample (2) Self-reports	(1) Viral load (2) Study questionnaire	(1) Dichotomous (2) Dichotomous	Inter-Instrument Reliability, within-rater reliability
Vreeman et al 2019^55^	HIV	Kenya, South Africa, Thailand	(1) Electronic record (2) Self-reports	(1) MEMS (2) Self-reported questionnaire	(1) Dichotomous (2) Dichotomous	Sensitivity
Wickersham et al 2018^56^ (Study 1)	HIV	United States	(1) Electronic record (2) Self-reports	(1) Electronic event monitoring (2) 9-item Morisky Medication Adherence Scale	(1) Numerical (2) Numerical	Convergent Validity, Concurrent Validity, Content Validity, Test-Retest Reliability, Internal Consistency Reliability
Wickersham et al 2018^56^ (Study 2)	HIV	United States	(1) Electronic record (2) Self-reports	(1) Electronic event monitoring (2) 9-item Morisky Medication Adherence Scale	(1) Numerical (2) Numerical	Convergent Validity, Concurrent Validity, Content Validity, Test-Retest Reliability, Internal Consistency Reliability
Wilson et al 2016^57^	HIV	United States	(1) Electronic record (2) Self-reports	(1) Medication event monitoring system (2) Study questionnaire	(1) Numerical (2) Numerical	Internal Consistency Reliability
Zhang et al 2020^58^	HIV	China	(1) Hair sample (2) Self-reports (3) Self-reports (4) Self-reports (5) Self-reports	(1) Hair tenofovir (2) Frequency of adherence behavior questionnaire (3) Percent of days of adherence questionnaire (4) Visual analog scale (5) Composite adherence scores	(1) Dichotomous (2) Ranked ordinal (3) Numerical (4) Numerical (5) Dichotomous	Internal-instrument reliability
Zissette et al 2021^59^	HIV	South Africa, Kenya	(1) Self-reports	(1) Monitoring tool	(1) N/A	Content Validity, Internal Consistency Reliability
Alhazzani et al 2021^60^	Diabetes	Saudi Arabia	(1) Self-reports (2) Self-reports	(1) English Self-Efficacy for Appropriate Medication Use Scale (2) Arabic Self-Efficacy for Appropriate Medication Use Scale	(1) Numerical (2) Numerical	Discriminant Validity, Construct Validity, Test-Retest Reliability, Internal Consistency Reliability
Anuradha, Prabhu, and Kalra 2022^61^	Diabetes	India	(1) Self-reports	(1) Self-reported questionnaire	(1) N/A	Internal Consistency Reliability, Inter-Item Reliability
Ashur et al 2015^62^	Diabetes	Libya	(1) Blood sample (2) Self-reports (3) Self-reports	(1) HbA1c (2) English 8-item Morisky Medication Adherence Scale questionnaire (3) Arabic 8-item Morisky Medication Adherence Scale questionnaire	(1) Dichotomous (2) Ranked ordinal (3) Ranked ordinal	Internal Consistency Reliability, Split-Half Reliability, Positive Predictive Value, Negative Predictive Value, Specificity, Sensitivity
Athavale et al 2019^63^	Diabetes	United States	(1) Pharmacy record (2) Self-reports (3) Self-reports (4) Self-reports	(1) Proportion of days covered (2) 5-point 1986 Morisky scale (3) Medication Adherence Reasons Scale (4) Medication adherence Estimation and Differentiation Scale	(1) Numerical (2) Numerical (3) Numerical (4) Numerical	Convergent Validity, Discriminant Validity, Internal Consistency Reliability
Ayoub et al 2019^64^	Diabetes	Lebanon	(1) Self-reports (2) Self-reports	(1) Lebanese Medication Adherence Scale (2) Diabetes Medication Adherence Scale	(1) Dichotomous (2) Dichotomous	Convergent Validity, Positive Predictive Value, Negative Predictive Value, Specificity, Sensitivity, Internal Consistency Reliability, Inter-Instrument Reliability
Bailey et al 2014^65^	Diabetes	United States	(1) Electronic record (2) Electronic record	(1) YSI 2300 STAT Plus glucose and lactate analyzer (2) Enlite Numerical glucose monitoring	(1) Dichotomous(2) Dichotomous	Uncategorized Validity
Barola et al 2021^66^	Diabetes	India	(1) Blood sample (2) Self-reports	(1) HbA1c (2) Hindi Diabetes Self-Management Profile Self Report	(1) Dichotomous (2) Numerical	Structural Validity, Internal Consistency Reliability, Predictive Validity
Boettcher et al 2015^67^	Diabetes	Austria, Germany	(1) Blood sample (2) Electronic record	(1) HbA1c (2) Self-monitoring blood glucose meters	(1) Dichotomous (2) N/A	Uncategorized Validity
Borot et al 2014^68^ (Study 1)	Diabetes	France	(1) Electronic record	(1) JewelPUMP	(1) N/A	Uncategorized Validity
Borot et al 2014^68^ (Study 2)	Diabetes	France	(1) Electronic record	(1) Animas Vibe	(1) N/A	Uncategorized Validity
Borot et al 2014^68^ (Study 3)	Diabetes	France	(1) Electronic record	(1) Accu-Chek Combo	(1) N/A	Uncategorized Validity
Borot et al 2014^68^ (Study 4)	Diabetes	France	(1) Electronic record	(1) MiniMed Paradigm 712	(1) N/A	Uncategorized Validity
Borot et al 2014^68^ (Study 5)	Diabetes	France	(1) Electronic record	(1) OmniPod	(1) N/A	Uncategorized Validity
Chan et al 2020^69^	Diabetes	UK	(1) Self-reports (2) Self-reports	(1) Beliefs about Medicines Questionnaire (2) Medication Adherence Report Scale	(1) Numerical (2) Numerical	Internal Consistency Reliability, Construct Validity
Chung et al 2015^70^ (Study 1)	Diabetes	Malaysia	(1) Blood sample (2) Self-reports	(1) HbA1c (2) Malaysian Medication Adherence Scale	(1) Dichotomous (2) Ranked ordinal	Sensitivity, Specificity, Negative Predictive Value, Positive Predictive Value, Concurrent Validity
Chung et al 2015^70^ (Study 2)	Diabetes	Malaysia	(1) Self-reports (2) Self-reports	(1) 8-item Morisky Medication Adherence Scale (2) Malaysian Medication Adherence Scale	(1) Ranked ordinal (2) Ranked ordinal	Internal Consistency Reliability, Test-Retest Reliability
Dibonaventura et al 2014^71^	Diabetes	United States	(1) Blood sample (2) Self-reports	(1) HbA1c (2) 8-item Morisky Medication Adherence Scale questionnaire	(1) Dichotomous (2) Ranked ordinal	Construct Validity, Internal Consistency Reliability
Edge et al 2017^72^	Diabetes	UK	(1) Blood sample (2) Electronic record	(1) Capillary blood glucose (2) Freestyle Libre sensor	(1) Dichotomous (2) N/A	Uncategorized Validity
Goh et al 2020^73^ (Study 1)	Diabetes	Malaysia	(1) Blood sample (2) Self-reports	(1) HbA1c (2) Patient Medication Adherence Instrument	(1) Dichotomous (2) Dichotomous	Construct Validity, Positive Predictive Value, Negative Predictive Value, Sensitivity, Specificity, Concurrent Validity, Internal Consistency Reliability
Goh et al 2020^73^ (Study 2)	Diabetes	Malaysia	(1) Blood sample (2) Self-reports	(1) HbA1c (2) Healthcare Professional Medication Adherence Instrument	(1) Dichotomous (2) Dichotomous	Construct Validity, Positive Predictive Value, Negative Predictive Value, Sensitivity, Specificity, Concurrent Validity, Internal Consistency Reliability
Gomes-Villas Boas, de Lima, and Pace 2014^74^	Diabetes	Brazil	(1) Self-reports (2) Self-reports	(1) Measurement of Adherence to Treatment - Oral Antidiabetics (2) Measurement of Adherence to Treatment - Insulin	(1) Numerical (2) Numerical	Face Validity, Criterion Validity, Sensitivity, Specificity, Internal Consistency Reliability, Uncategorized Reliability
Gonzalez et al 2013^75^	Diabetes	United States	(1) Blood sample (2) Electronic record (3) Self-reports	(1) HbA1c (2) MEMS (3) Self-reported questionnaire	(1) Dichotomous (2) Numerical (3) Numerical	Uncategorized Validity
Jansà et al 2013^76^	Diabetes	Spain	(1) Blood sample (2) Self-reports	(1) HbA1c (2) Diabetes Self-Care Inventory-Revised Version	(1) Dichotomous (2) Numerical	Internal Consistency Reliability, Test-Retest Reliability, Structural Validity
Kim et al 2016^77^	Diabetes	South Korea	(1) Blood sample (2) Self-reports (3) Self-reports (4) Self-reports	(1) HbA1c (2) Adherence to Refills and Medication Scale (3) Korean 8-item Morisky Medication Adherence Scale (4) Korean Adherence to Refills and Medication Scale	(1) Dichotomous (2) Dichotomous (3) Ranked ordinal (4) Dichotomous	Construct Validity, Convergent Validity, Known-Groups Validity, Internal Consistency Reliability
Kristina et al 2019^78^	Diabetes	Indonesia	(1) Blood sample (2) Self-reports (3) Self-reports	(1) Fasting blood glucose level (2) Medication Adherence Reasons Scale (3) Morisky Green Levine Medication Adherence Scale	(1) Dichotomous (2) Ranked ordinal (3) Ranked ordinal	Convergent Validity, Sensitivity, Specificity, Positive Predictive Value, Negative Predictive Value, Internal Consistency Reliability, Test-Retest Reliability
Laghousi et al 2021^79^	Diabetes	Iran	(1) Self-reports	(1) Persian 8-item Morisky Medication Adherence Scale questionnaire	(1) Ranked ordinal	Content Validity, Construct Validity, Internal Consistency Reliability, Test-Retest Reliability
Lai, Sellappans, and Chua 2020^80^	Diabetes	Malaysia	(1) Self-reports (2) Self-reports	(1) English Malaysian Medication Adherence Scale (2) Malay Malaysian Medication Adherence Scale	(1) Ranked ordinal (2) Dichotomous	Sensitivity, Specificity, Negative Predictive Value, Positive Predictive Value, Convergent Validity, Internal Consistency Reliability
Lee et al 2013^81^	Diabetes	Korea	(1) Blood sample (2) Self-reports (3) Self-reports	(1) HbA1c (2) 4-item Morisky Medication Adherence Scale questionnaire (3) Korean 4-item Morisky Medication Adherence Scale questionnaire	(1) Dichotomous (2) N/A (3) Ranked ordinal	Convergent Validity, Construct Validity, Sensitivity, Specificity, Negative Predictive Value, Positive Predictive Value, Test-Retest Reliability, Internal Consistency Reliability
Mallah et al 2019^82^	Diabetes	Lebanon	(1) Blood sample (2) Self-reports (3) Self-reports	(1) HbA1c (2) Lebanese Medication Adherence Scale (3) Diabetes Medication Adherence Scale	(1) Dichotomous (2) N/A (3) Dichotomous	Convergent Validity, Specificity, Sensitivity, Internal Consistency Reliability
Matsumoto et al 2021^83^	Diabetes	Japan	(1) Medical record (2) Pharmacy record (3) Self-reports	(1) Medical health insurance claims (2) Pharmacy insurance claims (3) Self-reported questionnaire	(1) N/A (2) N/A (3) N/A	Sensitivity, Specificity, Inter-Instrument Reliability
Matuleviciene et al 2014^84^	Diabetes	Sweden	(1) Electronic record (2) Electronic record	(1) DexcomG4 Sensor (2) Enlite Sensor	(1) Dichotomous (2) Dichotomous	Uncategorized Validity
Mayberry et al 2013^85^	Diabetes	United States	(1) Blood sample (2) Self-reports (3) Self-reports (4) Self-reports	(1) HbA1c (2) Summary of Diabetes Self-Care Activities medications subscale (3) Diabetes treatment satisfaction questionnaire (4) Adherence to Refills and Medications Scale (diabetes)	(1) Dichotomous (2) Numerical (3) Numerical (4) Numerical	Convergent Validity, Construct Validity, Internal Consistency Reliability, Predictive Validity
Mehta et al 2015^86^	Diabetes	United States	(1) Blood sample (2) Electronic record (3) Self-reports	(1) HbA1c (2) Blood glucose monitor (3) Diabetes Medication Questionnaire	(1) Dichotomous (2) N/A (3) Numerical	Convergent Validity, Criterion Validity, Internal Consistency Reliability, Test-Retest Reliability
Mikhael et al 2019^87^ (Study 1)	Diabetes	Iraq	(1) Blood sample (2) Self-reports (3) Self-reports	(1) HbA1c (2) Medication Adherence Questionnaire (3) Iraqi Anti-Diabetic Medication Adherence Scale	(1) Dichotomous (2) N/A (3) Ranked ordinal	Negative Predictive Value, Positive Predictive Value, Sensitivity, Specificity, Convergent Validity, Concurrent Validity, Internal Consistency Reliability, Test-Retest Reliability
Mikhael et al 2019^87^ (Study 2)	Diabetes	Iraq	(1) Blood sample (2) Self-reports	(1) HbA1c (2) Medication Adherence Questionnaire	(1) Dichotomous (2) N/A	Negative Predictive Value, Positive Predictive Value, Sensitivity, Specificity, Internal Consistency Reliability
Oliveira et al 2022^88^	Diabetes	Brazil	(1) Self-reports	(1) Self-reported questionnaire	(1) N/A	Content Validity, Inter-Instrument Reliability
Osborn and Gonzalez 2016^89^ (Study 1)	Diabetes	United States	(1) Self-reports (2) Self-reports	(1) Adherence to Refills and Medication Scale for Diabetes (2) Adapted Morisky Medication Adherence Scale for insulin adherence	(1) Dichotomous (2) Dichotomous	Convergent Validity, Criterion Validity, Internal Consistency Reliability
Osborn and Gonzalez 2016^89^ (Study 2)	Diabetes	United States	(1) Self-reports (2) Self-reports	(1) Summary of Diabetes Self-Care Activities medications subscale (2) Adapted Morisky Medication Adherence Scale for insulin adherence	(1) Dichotomous (2) Numerical	Convergent Validity
Osborn and Gonzalez 2016^89^ (Study 3)	Diabetes	United States	(1) Self-reports (2) Self-reports	(1) Summary of Diabetes Self-Care Activities insulin specific subscale (2) Adapted Morisky Medication Adherence Scale for insulin adherence	(1) Dichotomous (2) Numerical	Convergent Validity
Patton et al 2013^90^	Diabetes	United States	(1) Blood sample (2) Electronic record (3) Electronic record	(1) HbA1c (2) Blood glucose monitor (3) Bolus	(1) N/A (2) N/A (3) N/A	Inter-Instrument Reliability, Uncategorized Reliability
Ranasinghe et al 2018^91^	Diabetes	Sri Lanka	(1) Blood sample (2) Self-reports	(1) HbA1c (2) Brief medication questionnaire	(1) Dichotomous (2) Ranked ordinal	Criterion Validity, Specificity, Sensitivity, Test-Retest Reliability, Internal Consistency Reliability
Ratanawongsa et al 2015^92^	Diabetes	United States	(1) Blood sample (2) Blood sample (3) Blood sample (4) Blood sample (5) Pharmacy record	(1) HbA1c (2) Systolic blood pressure (3) diastolic blood pressure (4) Low-density lipoprotein (5) Numerical medication gap	(1) Dichotomous (2) Dichotomous (3) Dichotomous (4) Dichotomous (5) Dichotomous	Uncategorized Validity
Shi et al 2021^93^	Diabetes	China	(1) Self-reports (2) Self-reports (3) Self-reports	(1) 8-item Morisky Medication Adherence Scale questionnaire (2) General Adherence Scale (3) Chinese General Adherence Scale	(1) Ranked ordinal (2) N/A (3) N/A	Construct Validity, Content Validity, Criterion Validity, Internal Consistency Reliability, Split-Half Reliability, Test-Retest Reliability
Surekha et al 2016^94^	Diabetes	India	(1) Pill count (non-self-reported) (2) Self-reports	(1) In-clinic pill count (2) 8-item Morisky Medication Adherence Scale questionnaire	(1) Numerical (2) Ranked ordinal	Sensitivity, Specificity, Negative Predictive Value, Positive Predictive Value, Internal Consistency Reliability
Tandon et al 2015^95^	Diabetes	Togo	(1) Self-reports (2) Blood sample (3) Self-reports	(1) 4-item Morisky Medication Adherence Scale questionnaire (2) Fasting blood glucose level (3) 8-item Morisky Medication Adherence Scale questionnaire	(1) Ranked ordinal (2) Dichotomous (3) Ranked ordinal	Convergent Validity, Known-Groups Validity, Sensitivity, Specificity, Positive Predictive Value, Negative Predictive Value, Internal Consistency Reliability
Vincze, Losonczi, and Stauder 2020^96^	Diabetes	Hungary	(1) Blood sample (2) Self-reports (3) Self-reports	(1) HbA1c (2) 8-item Morisky Medication Adherence Scale questionnaire (3) Hungarian Diabetes Self-Management Questionnaire	(1) Dichotomous (2) Ranked ordinal (3) Numerical	Construct Validity, known-group validity
Wang et al 2012^97^	Diabetes	Singapore	(1) Self-reports	(1) Modified Morisky, Green, Levine Adherence Scale	(1) Numerical	Content Validity, Internal Consistency Reliability
Zongo et al 2016^98^	Diabetes	Canada	(1) Blood sample (2) Self-reports (3) Self-reports (4) Self-reports (5) Self-reports	(1) HbA1c (2) Self-report with 4 items (3) 8-item Morisky Medication Adherence Scale questionnaire (4) Proportion of missed pills (5) Single-item scale	(1) Dichotomous (2) Ranked ordinal (3) Ranked ordinal (4) N/A (5) N/A	Content Validity
Zongo et al 2016^99^	Diabetes	Canada	(1) Self-reports (2) Self-reports	(1) English 8-item Morisky Medication Adherence Scale questionnaire (2) French 8-item Morisky Medication Adherence Scale questionnaire	(1) Ranked ordinal (2) Ranked ordinal	Content Validity, Internal Consistency Reliability
Zongo et al 2019^26^	Diabetes	Canada	(1) Medical record (2) Pharmacy record (3) Pharmacy record	(1) Hospitalization records (2) Proportion of days covered (3) Daily polypharmacy possession ratio	(1) Dichotomous (2) Numerical (3) Numerical	Content Validity

Overall, 36 studies addressed adherence to HIV medications, 51 studies examined adherence to diabetes medications, and one study explored adherence to nutritional supplements. The highest percentage of studies focused on North America (32%) followed by East Asia and Pacific (20%), and Sub-Saharan Africa (16%). Most studies (73%) explored the psychometric properties of adherence as a primary aim, while 27% investigated the psychometric properties of adherence as a secondary aim.

### Measurement Modalities and Measures

We identified 9 modalities, 221 total measures, and 143 unique measures across all studies. The nine modalities included blood samples, hair samples, urine samples, electronic records, pharmacy records, medical records, pill counts (non-self-reported), self-reports (including questionnaires and pill counts), and other. Overall and within the HIV and diabetes studies, the three most common modalities were self-reports, blood samples, and electronic records, in that order (Table 2). Within the self-report modality, 97% of measures were questionnaires. The 8-item Morisky Medication Adherence Scale (MMAS-8) was the most frequently used self-reported measure, with 14 instances of use. Among the blood sample measures, 40% tested HbA1c levels and 25% examined viral loads.

**Table 2:** Distribution of Measures and Modalities by Health Area

Modality Category	Modality	Number of Measures			
		HIV	Diabetes	Nutrition	All
	**Self-reports**	48 (48.5%)	67 (56.8%)	1 (25%)	116 (52.5%)
	**Pill Count (non-self-reported)**	9 (9.1%)	1 (0.8%)	1 (25%)	11 (5.0%)
	**Other**	1 (1.0%)	0 (0.0%)	0 (0.0%)	1 (0.4%)
**Biometric Samples**	**Blood Sample**	24 (24.2%)	28 (23.7%)	1 (25%)	53 (24.0%)
	**Hair Sample**	2 (2.0%)	0 (0.0%)	0 (0.0%)	2 (0.9%)
	**Urine Sample**	1 (1.0%)	0 (0.0%)	0 (0.0%)	1 (0.4%)
**Written Records**	**Electronic Record**	10 (10.1%)	15 (12.7%)	1 (25%)	26 (11.8%)
	**Pharmacy Record**	3 (3.0%)	5 (4.2%)	0 (0.0%)	8 (3.6%)
	**Medical Record**	1 (1.0%)	2 (1.7%)	0 (0.0%)	3 (1.4%)
	**TOTAL**	99 (100%)	118 (100%)	4 (100%)	221 (100%)

**Figure 2:**
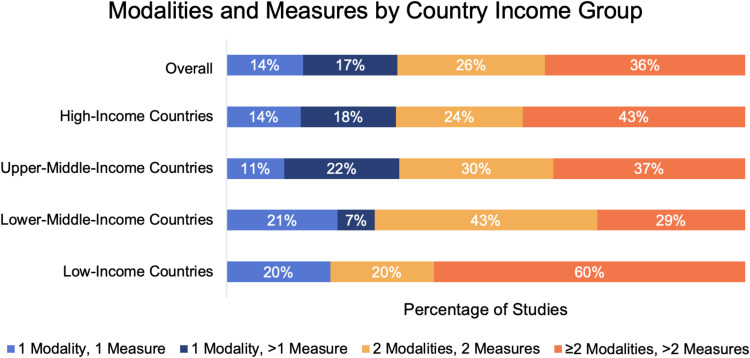
Modalities And Measures, By Country Income Group.

By country income groups, 49 studies were conducted in high-income countries (HICs), 27 studies in upper-middle-income countries (UMICs), 14 studies in lower-middle-income countries (LMICs), and 5 studies in low-income countries (LICs). (Seven studies in multiple country income groups were double counted). The use of triangulation was similar across country income groups. In each group, more than half the studies included at least two types of data collection methods and two different measures (Figure 2). The mean number of modalities in each study was 1.84 (SD = 0.73) overall, 1.90 (SD = 1.17) for HICs, 1.85 (SD = 1.18) for UMICs, 1.86 (SD = 0.86) for LMICs, and 1.8 (SD = 1.14) for LICs. In HICs, UMICs, and LICs, studies with more than two modalities and more than two measures were most common, whereas in LMICs, studies with exactly two modalities and two measures were most common. Almost no relationship exists between country income level and triangulation.

### Adherence Definitions

The operational definition of adherence fell into four categories: numerical, dichotomous, ranked ordinal, and undefined. Numerical definitions define adherence discretely (eg, scale scores) or continuously (eg, percentages). Examples of numerically defined adherence include percentages of pills taken in a certain time frame or adherence levels on visual analog scales in self-reported questionnaires. For example, Zhang et al asked participants how many days they took their medications as prescribed in the last month.^58^ From the responses, the authors calculated the percentage of days the participants were adherent in the past 30 days.^58^

Dichotomous definitions describe adherence in two states, such as adherent/non-adherent, good glycemic control/poor glycemic control, or undetectable viral load/detectable viral load. Cutoff points to dichotomize adherence across studies were the same for some measures such as HbA1c where good glycemic control was defined as HbA1c <7%. The threshold for adherence varied across studies for other measures such as viral load, where participants could be classified as “adherent” if their viral load was ≤20 copies/mL or ≤400 copies/mL depending on the study.

Finally, ranked ordinal definitions describe adherence as having multiple levels. Most measures (65%) defining adherence with a ranked ordinal scale used MMAS, which categorized adherence into high/medium/low.^62^,^70^,^71^,^77–81^,^93–96^,^98^,^99^ Based on the total score of the scale, high adherence was defined as a score of eight, medium as a score of six or seven, and low as a score less than six.^62^,^70^,^71^,^77–81^,^93–96^,^98^,^99^

Adherence was not defined for 40 (18%) measures. Approximately 14% of HIV measures, 21% of diabetes measures, and 25% of nutritional supplement measures did not define adherence. Many of the studies that did not define adherence aimed to create a new measure or to translate an existing measure. Some reasons why studies did not define adherence included having study aims testing the correlation between adherence measures,^24^,^28^,^41^,^45^,^46^,^67^,^81–83^,^90^,^101^ testing the internal reliability of a new measure,^53^,^59^,^61^ testing the content validity of a new measure,^53^,^61^,^88^ and testing the construct validity of a new measure.^53^,^59^

Overall, dichotomously defined measures were the most popular (89 of 221 measures or 40%) followed by numerical (27%) and ranked ordinal (14%). Dichotomous measures were also the most popular way to define adherence across health topics, country income levels, and measure types (direct/indirect). However, the most common type of adherence definition fluctuated across modalities because of the measures within the modality. For example, the dichotomous adherence definition type was most common within the blood sample modality because viral load and HbA1c measures define adherence dichotomously and comprise 64% of the blood samples in the review. Similarly, the majority of adherence definitions in the pharmacy modality were numerical because adherence was often expressed as a rate, such as the proportion of days covered or medication possession ratio.^27^,^31^,^34^,^63^,^102^

The amount of variation in an adherence definition category within a modality depended on 1) whether the measures within the modality could be assessed numerically and 2) whether widely accepted cutoffs existed for the measure. There was more variation in adherence definition types within modalities if measures could initially be measured numerically. This is because the researchers decided how they wanted to categorize adherence after collecting numeric data. For example, pill count measures were initially measured numerically. Some studies reported pill count as a discrete number or a percentage of remaining pills given the original number of pills dispensed.^24^,^26^,^27^,^29^,^32^,^35^,^37^,^38^,^43^,^47^,^50^,^94^ Other studies reported pill count in a dichotomous or ranked ordinal manner. For example, Teshome et al defined “high adherence” as the healthcare worker not seeing ≥80% of pills dispensed for the past 30 days.^24^

For self-reported measures, the wide variety of scales used across studies and the originally continuous nature of most scales resulted in a wide variation of adherence definitions within the modality. For example, Ayoub et al and Mallah et al were the only studies in the review to measure adherence using the Lebanese Medication Adherence Scale (LMAS-14), which initially measured adherence numerically on a scale from 0 to 42.^64^,^82^ Ayoub et al defined adherence on LMAS-14 dichotomously by classifying patients as adherent or non-adherent using a cut-off point of 38.^64^ Meanwhile, Mallah et al did not define adherence for LMAS-14 as the scale was a reference measure a new scale.^82^ Finally, there was less variation in adherence definition types within modalities if measures had widely accepted cutoffs, even if the measures could initially be defined numerically. For example, all measures for HbA1c in the review were originally measured numerically. However, adherence was reported dichotomously across studies, with good glycemic control as HbA1c <7%.^62^,^66^,^67^,^70^,^71^,^73^,^75–77^,^81^,^82^,^85–87^,^91^,^92^,^96^,^98^

### Reporting Validity/ Reliability

**Figure 3:**
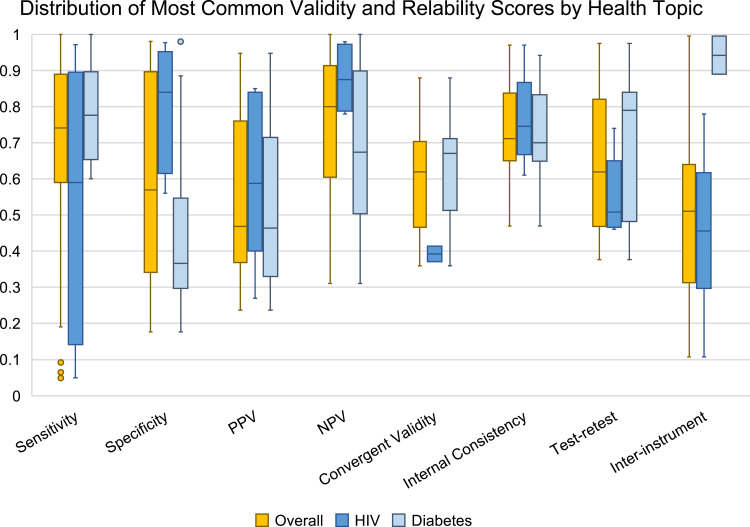
Distribution Of Select Validity and Reliability Scores, By Health Topic.

Among the studies in the review, 46 (52%) reported both validity and reliability, 28 studies (31%) only reported validity, and 14 studies (16%) only reported reliability. Fourteen types of validity and six types of reliability appeared across the studies (Table 3). Almost half of all studies measured internal consistency reliability (through Cronbach’s alpha), and more than a quarter of studies tested sensitivity and specificity. Scores for the five most common validity types and three most common reliability types had overall distributions of at least 0.5 across all studies. Overall, Cronbach’s alpha had the lowest score range of 0.5 (0.97 to 0.47) with a mean score of 0.76 (Figure 3). The next most common type of reliability measured in the review (test-retest reliability) had a mean score of 0.55 with a range from 0.376 to 0.975. Finally, the Kappa coefficient for inter-instrument reliability had a mean score of 0.49 with a range from 0.107 to 0.995. Of the five most common validity measures, the negative predictive value had the highest mean score (0.76, range: 0.311–1), followed by sensitivity (0.66, range: 0.049–1), specificity (0.60, range: 0.177–0.98), positive predictive value (0.55, range: 0.237–0.948), and convergent validity (0.53, range: 0.36–0.88).

**Table 3:** Types of Validity and Reliability

Validity		Reliability	
Validity Type	Number of studies (%)	Reliability Type	Number of studies (%)
Sensitivity	26 (29.5%)	Internal Consistency Reliability	41 (46.6%)
Specificity	25 (28.4%)		
Positive Predictive Value	17 (19.3%)		
Convergent Validity	17 (19.3%)	Test-retest Reliability	14 (15.9%)
Negative Predictive Value	16 (18.2%)		
Content Validity	12 (13.6%)	Inter-instrument Reliability	8 (9.09%)
Construct Validity	12 (13.6%)		
Criterion Validity	10 (11.4%)	Split-half Reliability	2 (2.3%)
Concurrent Validity	8 (9.1%)		
Predictive Validity	4 (4.5%)	Inter-item Reliability	1 (1.1%)
Known-groups Validity	2 (2.3%)		
Discriminant Validity	2 (2.3%)	Within-rater Reliability	1 (1.1%)
Structural Validity	2 (2.3%)		
Face Validity	1 (1.1%)	Uncategorized Reliability	12 (13.6%)
External Criterion Validity	1 (1.1%)		
Uncategorized Validity	15 (17.0%)

Within each type of validity and reliability, the distribution of scores varied greatly between HIV and diabetes studies, except for internal consistency (Figure 3). (Nutritional supplementation was excluded from Figure 3 because there was only one study included in the review). The range of validity/reliability scores for diabetes studies was the same or similar to the overall score ranges, except for sensitivity and inter-instrument reliability. The sensitivity range for diabetes studies is about half (0.4) of the overall range because the overall sensitivity includes outliers from HIV studies. The range of inter-instrument reliability scores was also smaller (0.105) for diabetes than for the overall range. Diabetes studies reported a greater number of psychometric scores across the most common validity/reliability categories in Figure 3, except for inter-instrument reliability. Only two inter-instrument reliability scores were reported across diabetes studies.

In HIV studies, the median scores for specificity, positive predictive values, and negative predictive values were higher compared to diabetes studies, whereas in diabetes studies, the median scores for sensitivity and convergent validity were higher compared to HIV studies. Additionally, across the three most common types of reliability, median scores were higher for diabetes studies compared to HIV studies, except for internal consistency.

Among HIV studies, about 30% explored only validity or reliability, and 39% explored both validity and reliability. Most (61%) diabetes studies examined both validity and reliability, and the nutritional supplement study examined both psychometric properties as well. For UMICs, LMICs, and LICs, studies reporting both validity and reliability were most common. Studies only investigating validity were most common for HICs. Additionally, most studies explored both validity and reliability in all regions except Europe (where studies exploring only validity were most common).

### True Effect Sizes

Of the 46 studies that reported validity and reliability, 26 shared a Pearson’s correlation coefficient, indicating the strength of the linear relationship between the test and reference measures used to measure adherence in each study. Studies that reported these results were nearly evenly divided between diabetes and HIV studies (57% and 43%, respectively). Pearson r values, which in these studies were calculated to show the correlation between test and reference measures, ranged from 0.09–0.93. From those values, we grouped studies into “low” (0.09–0.36), “medium” (0.37–0.65), or “high” (0.66–0.03) effect size. Some studies reported multiple effect sizes, using different reference and test measures, so those effect sizes have been included to reach a total of 28 reported effect sizes. Overall, we found that seven studies fell into the “low” range, 11 in the “medium” range, and 13 in the “high” effect size range. There was no significant difference found in whether these studies were reporting on adherence to diabetes or HIV medication; whether they were conducted in high-, middle-, or low-income settings; or based on population size.

## Discussion

In this review, we summarized the methods and modalities used to measure adherence to HIV medication, diabetes medication, and nutritional supplements. We also analyzed variations in adherence definitions and how the psychometric properties are measured and reported.

Across 88 studies in the review, there were 9 modalities, 221 total measures, and 143 unique measures. All modalities and measures have strengths and limitations related to their acceptability, feasibility, reliability, and validity. Through triangulation, researchers can compare modalities and measures to choose those that best suit their study needs. In practice, triangulating modalities or measures lead to more flexibility in the field. Instead of relying on one measure, such as a medication event monitoring system, which requires specific equipment and training, having multiple acceptable adherence measures or modalities grants more feasibility for adherence research in a variety of contexts, especially in low-resource settings. Some measures may also overestimate or underestimate adherence systematically, while others may do so randomly. If the same measure is used in all studies, researchers would replicate the same limitation associated with the measure or modality across studies. Researchers can better balance measurement errors across adherence measures by using some that are more likely to overestimate and underestimate adherence to more accurately measure adherence behavior. Therefore, since no gold standard adherence measure exists, we recommend researchers include multiple measures and modalities in their studies and use triangulation to balance individual measurement errors and leveraging the diverse strengths of various measures and modalities.

In the review, we also identified three types of operational definitions for adherence: numerical, dichotomous, and ranked ordinal. While categorizing patients into levels of adherence can help clinicians or researchers divide patients for treatment interventions, follow-up, or other actions, the scientific significance of a cutoff is often marginal. Someone who is 94% adherent or 96% adherence on the scale may not have different health outcomes even if they are categorized as having poor adherence and good adherence respectively. Similarly, many studies measuring viral load defined dichotomous adherence cutoffs based on the test’s sensitivity or limit of detection rather than differences in clinical outcomes.^29^,^33^,^35^,^42^,^44^,^46^,^47^,^49^,^50^,^54^ This is why in some studies using sensitive instruments to measure viral load, undetectable viral load/adherence is classified as ≤20 copies/mL whereas in other studies using less sensitive instruments, undetectable viral load/adherence is classified as ≤400 copies/mL. One solution to these cutoffs is to conceptualize adherence as a spectrum and report adherence numerically.

Finally, the limited number of statistics for one type of validity or reliability in the review restricted our results to be largely qualitative and hindered our ability to compare statistical values. To allow for future quantitative reviews of the psychometric properties of validity and reliability, researchers could report a core group of statistics across studies. Future research and discussions are needed to determine which measures would be most meaningful and feasible to include in the core statistics group.

The distribution of scores within one type of validity or reliability measure differed greatly between HIV and diabetes, except for internal consistency. One reason for this difference among validity measures could be the nature of the diseases. HIV is an infectious disease, while diabetes is a non-communicable disease. The median scores and ranges for negative predictive value and specificity were higher than positive predictive value and sensitivity. This indicates that when choosing adherence measures for HIV, researchers prioritize minimizing false positives and maximizing true negatives over minimizing false negatives and maximizing true positives. In other words, it is more important to correctly identify people who are non-adherent than people who are adherent for HIV. This could be because people who are not virally suppressed may transmit the disease to others and are more susceptible to other diseases.

Finally, when calculating true effect sizes between the reference and test adherence measures, we found that 7 studies fell into the “low” range, 11 in the “medium” range, and 13 in the “high” effect size range. The higher effect size suggests more agreement between the reference adherence measure and the measure being tested. The variability in effect size among studies in our review shows a lack of consistency in the strength of the relationships between reference and test adherence measurements. Therefore, we are likely still far from finding a “gold standard” approach to adherence measurement.

## Limitations

One limitation is the exclusion of non-English language studies, which would have provided more insights into the psychometric properties of adherence measures. Additionally, restricting the search to peer-reviewed primary research studies published within a ten-year period might have overlooked relevant literature published before or after this timeframe, potentially leading to gaps in the understanding of adherence. Expanding the timeframe searched would have improved our ability to present an unbiased summary of the psychometric properties of adherence within the HIV, diabetes, and nutritional supplementation literature. Finally, only one reviewer looked at all potential studies and analyzed the data, which limited the robustness of the review by constraining the diversity of perspectives in the analysis.

## Conclusion

Measuring adherence accurately and reliably continues to be a challenge for research in HIV, diabetes, and nutritional supplementation. The ability to accurately measure adherence is imperative to assessing and monitoring the health of people with chronic diseases and reducing their morbidity and mortality. Currently, there is no standard operational definition for adherence or a widely accepted adherence measure for HIV, diabetes, or nutritional supplementation. Instead of searching for a standard measure, a rigorous way to measure adherence could be through multiple modalities and measures that all triangulate to a common conclusion.

### Recommendations for Reporting

Based on the findings in our review, we offer three key recommendations for reporting adherence measurements. First, researchers should report adherence results from multiple measures and modalities. Each measurement and modality type has different strengths and limitations, which triangulation can help balance. In the absence of a gold standard measure, using multiple measures and cross-checking results can help enhance the validity of research findings and mitigate bias and provide a practical and nuanced solution to measuring adherence. Second, adherence results should be reported numerically. While categorizing patients into different adherence categories may be helpful in clinical settings, the cutoffs for categorization can differ based on a test’s precision and may not reflect differences in clinical outcomes. If researchers feel that categorization is appropriate, we still recommend reporting numeric results in case cutoffs change in the future. Finally, researchers investigating the validity and reliability of adherence measures should report multiple types of validity and reliability in their studies (including Cronbach’s alpha) to improve statistical comparisons across adherence measures.
